# Acoustic and Clinical Data Analysis of Vocal Recordings: Pandemic Insights and Lessons

**DOI:** 10.3390/diagnostics14202273

**Published:** 2024-10-12

**Authors:** Pedro Carreiro-Martins, Paulo Paixão, Iolanda Caires, Pedro Matias, Hugo Gamboa, Filipe Soares, Pedro Gomez, Joana Sousa, Nuno Neuparth

**Affiliations:** 1 Comprehensive Health Research Center (CHRC), LA-REAL, NOVA Medical School, Campo Mártires da Pátria, 130, 1169-056 Lisboa, Portugalnneuparth@gmail.com (N.N.); 2 Serviço de Imunoalergologia, Hospital de Dona Estefânia, ULS São José, Rua Jacinta Marto, 1169-045 Lisbon, Portugal; 3 Fraunhofer Portugal AICOS, Rua Alfredo Allen 455/461, 4200-135 Porto, Portugalfilipe.soares@aicos.fraunhofer.pt (F.S.); 4 Laboratory for Instrumentation, Biomedical Engineering and Radiation Physics (LIBPhys), Faculdade de Ciências e Tecnologia, NOVA University of Lisbon, Caparica, 2820-001 Lisbon, Portugal; 5 NeuSpeLab, CTB, Universidad Politécnica de Madrid, Campus de Montegancedo, s/n, 28223 Madrid, Spain; pedrogvilda@telefonica.net; 6 NOS Inovação, Rua Actor António Silva, 9–6° Piso, Campo Grande, 1600-404 Lisboa, Portugal

**Keywords:** diagnostic tests, machine learning, SARS-CoV-2, speech, voice

## Abstract

**Background/Objectives:** The interest in processing human speech and other human-generated audio signals as a diagnostic tool has increased due to the COVID-19 pandemic. The project OSCAR (vOice Screening of CoronA viRus) aimed to develop an algorithm to screen for COVID-19 using a dataset of Portuguese participants with voice recordings and clinical data. **Methods:** This cross-sectional study aimed to characterise the pattern of sounds produced by the vocal apparatus in patients with SARS-CoV-2 infection documented by a positive RT-PCR test, and to develop and validate a screening algorithm. In Phase II, the algorithm developed in Phase I was tested in a real-world setting. **Results:** In Phase I, after filtering, the training group consisted of 166 subjects who were effectively available to train the classification model (34.3% SARS-CoV-2 positive/65.7% SARS-CoV-2 negative). Phase II enrolled 58 participants (69.0% SARS-CoV-2 positive/31.0% SARS-CoV-2 negative). The final model achieved a sensitivity of 85%, a specificity of 88.9%, and an F1-score of 84.7%, suggesting voice screening algorithms as an attractive strategy for COVID-19 diagnosis. **Conclusions:** Our findings highlight the potential of a voice-based detection strategy as an alternative method for respiratory tract screening.

## 1. Introduction

Diseases of the respiratory tract frequently affect the vocal apparatus, which leads to changes in the timbre of the voice.

Analysing the voice and other audio signals is an attractive tool for screening diseases, as sound recordings are easy to obtain and non-invasive [1,2,3]. However, due to the subtle changes in voice and cough characteristics, artificial intelligence techniques are required to recognise specific disease patterns [4]. Especially for biomarkers derived from audio recordings, these patterns may present geographical, linguistic, gender, and age-related differ-ences, which makes this problem more challenging than it could be at first glance.

Interest in this topic has also increased as a result of the COVID-19 pandemic [5,6,7], during which, some research groups have tried to develop more convenient and accessible diagnostic methods by using machine learning (ML) and deep learning (DL) to analyse voice recordings and other audio signals such as coughing, breathing sounds, and respiratory rate [5,6].

However, most algorithms have used crowdsourced databases, which do not guarantee the quality of the recordings and only contain a limited number of positive samples. The most commonly used and well-known respiratory audio-based datasets include the Coswara dataset [8], created as part of the Coswara Project developed by the Indian Institute of Science Bangalore, and the COVID-19 Sounds database [9], the largest multimodal dataset of respiratory sounds. These two datasets were made available (the latter upon request) to the research community.

Research groups have continuously explored ML and DL methodologies for COVID-19 detection using cough, breathing, and speech recordings to capture changes in the vocal tract of infected subjects. Brown et al. [10] proposed a machine learning (ML) pipeline that extracts features from audio recordings of coughing and breathing (from the COVID-19 Sounds database) and feeds them into a statistical model to distinguish between cases with COVID-19 and without COVID-19. Their best results achieved a specificity of 80% and a sensitivity of 72%. Anupam et al. [11] implemented their COVID-19 screening framework by analysing cough recordings from a subset of the Coswara database. The processing steps included audio cleaning, feature extraction, and classification. The best result was obtained with a Support Vector Machine (SVM) model, which had a sensitivity of 97% and a specificity of 98%.

Coppock et al. [12] developed a custom convolutional neural network (CNN) to analyse spectrograms of cough and breathing audios from COVID-19 Sounds, achieving 90.9% area under the receiver operating characteristic curve (AUROC) and 77.4% sensitivity in discriminating COVID-19 positive cases from those with asthma. A similar study was conducted by Pahar et al. [13] that compared classical ML and deep neural network (DNN) models using raw cough audio features from Coswara and Sarcos databases, with a ResNet50 model achieving 98% and 57% specificity and 93% sensitivity, respectively. Melek et al. [14] studied the detection of COVID-19 from single cough sounds using Virufy and NoCoCoDa datasets, finding a K-nearest neighbors model yielding around 94% accuracy. Meister et al. [15] conducted an extensive feature ranking across Coswara and COVID-19 Sounds audio data, highlighting the importance of spectral and cepstral features, with a Random Forest model achieving around 87% AUROC.

Nevertheless, the validity of these previous results is compromised by the unreliable data sources containing either unconfirmed COVID-19 infection labels or uncontrolled recording frameworks. Therefore, some researchers have taken it upon themselves to create a recording protocol that ensures the reliability of their results [16]. For instance, using an RBF-SVM model, Robotti et al. [17] employed multiple vocal data sources (including vowel phonation, speech, and cough) to execute a series of feature extraction, selection, and classification steps. The authors achieved an average accuracy of 80% using data recorded under controlled settings. Similarly, Costantini et al. [18] applied two custom ML (AdaBoost classifier) and DL (CNNs) algorithms to identify COVID-19 infection using a pool of 310 recruited individuals. The authors tried to distinguish between COVID-19 infected/recovered patients and healthy controls in two separate classification tasks, having obtained an accuracy of 100% and 86.1%, respectively. An identical data collection study by Hassan et al. [19] used a DNN model to analyse cough, breathing, and voice samples, achieving 98% precision and 100% sensitivity only using breathing recordings. Modi et al. [20] also carried out a data collection procedure, recording vocal samples from 117 COVID-19 infected patients and healthy controls. Short-time Fourier representations were used as features to feed a logistic regression algorithm, resulting in an average file-level accuracy of 74.80%. This helps support the idea that high-quality datasets (obtained with validated labels and in controlled settings) lead to better outcomes, either in terms of performance results or even the meaningfulness of insights captured from data. Nonetheless, most studies still lack a robust assessment of the validity and quality of the audios recorded before undergoing further processing stages, in order to increase confidence in the obtained outcomes.

Unlike the studies described in this section, our proposed framework relies on a more robust data curation pipeline executed before extracting features and training ML models. This involves: (i) recording high-quality uncompressed audio, (ii) discarding silent portions of each audio, and (iii) validating the quality of the audios and their relevant segments using pre-trained DL models.

This paper presents the dataset and the results of the OSCAR (vOice Screening of CoronA viRus) project. This study aimed to develop an algorithm for early screening of COVID-19 and test it in a real-world subject scenario. The OSCAR dataset is the first audio dataset collected in Portugal, combining (a) audio recording exercises, (b) self-reported data, and (c) validated labels of SARS-CoV-2 infection, providing a robust resource for testing screening algorithms of COVID-19 or other respiratory diseases. By combining a carefully designed audio processing pipeline, meaningful features, and a reliable source of data containing validated SARS-CoV-2 infection labels, we believe the presented outcomes may support future validation trials and foster the integration of digital screening tools into clinical practice.

## 2. Methods

### 2.1. Study Design, Setting, and Participants

The OSCAR project was a cross-sectional observational study in Portugal divided into two phases.

Phase I aimed to characterise the pattern of sounds produced by the vocal apparatus in patients with SARS-CoV-2 infection documented by a positive RT-PCR test to develop and validate an algorithm. Participant recruitment took place from 1 June 2021 to 8 April 2022 at four centres: Centro de Medicina Laboratorial Germano de Sousa, Hospital da Luz—Lisbon, Hospital Beatriz Ângelo, and NOVA Medical School. A team member from each recruitment centre extended the invitation to participate. For this purpose, a leaflet with a Q.R. code and a unique participant code was distributed.

To be eligible, participants had to be ≥18 years of age, be able to read, understand, and speak Portuguese, have a nasopharyngeal swab taken to identify SARS-CoV-2 using RT-PCR (real-time protein chain reaction), have access to a computer/smartphone or tablet, and have an internet connection to answer the online questionnaire and complete the requested audio recording. Exclusion criteria were the inability to understand the consent form, produce vocal sounds, or articulate small sentences in Portuguese.

Phase II (from 19 January to 31 May 2022) tested the algorithm developed in Phase I in a real-world setting. In this Phase, the general population could participate, and invitations were made through the project partners’ social networks, allowing for a more diverse and broader sample. The inclusion and exclusion criteria were similar to Phase I, but RT-PCR and TRAg (rapid antigen test) were eligible for participation. Convenience sampling was used in Phases I and II.

This study was approved by the Ethics Committee of NOVA Medical School/Faculdade de Ciências Médicas, Universidade Nova de Lisboa (78/2021/CEFCM, 31 May 2021) and by the Ethics Committees of the different recruitment centres. Participants were informed about the study, and their digitally signed consent was obtained according to Portuguese laws and regulations.

### 2.2. Data Collection

**Figure 1:**
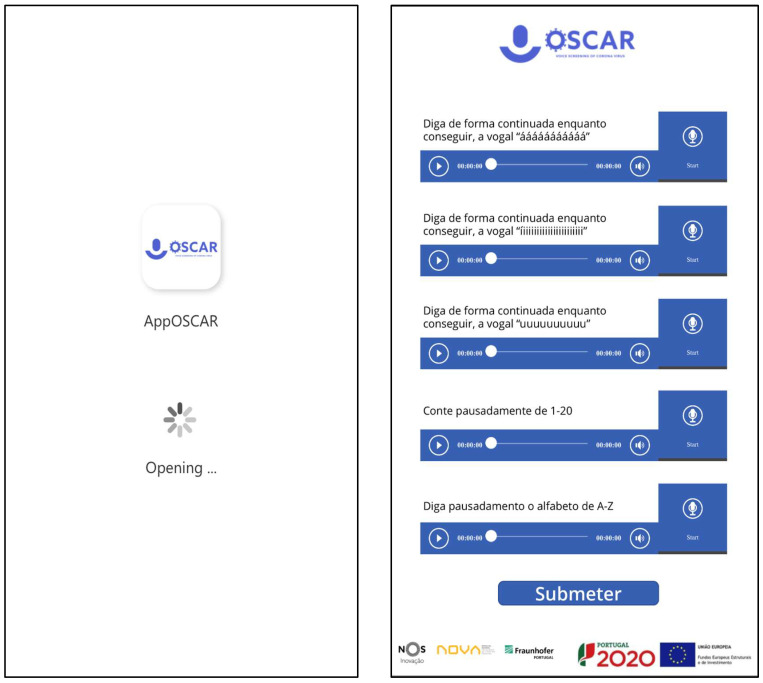
Data collection user-interface. The (**left**) screen outlines the application initialisation. The (**right**) screen shows up upon the collection of audio samples.

Data were collected through an online form created by the study team (see Figure 1). Participants independently accessed the study link and answered the questionnaire after signing the digital informed consent form (DocuSign, Inc., San Francisco, CA, USA). The data collection comprised two stages: (1) self-reported data, such as sociodemographic information (gender, age, country, and region), clinical data (symptoms and prior illnesses) and the RT-PCR/TRAg test result for SARS-CoV-2 (collected previously), and (2) vocal data, obtained through exercises of (a) breathing, (b) coughing, and (c) vocalisation of vowels and phrases (previously defined in the form). For the development of the algorithm, only the responses and vocal recordings given up to five days after performing the SARS-CoV-2 test were considered. European Union General Data Protection and Regulation (GDPR) privacy laws were respected.

### 2.3. Audio Quality Assessment

**Figure 2:**
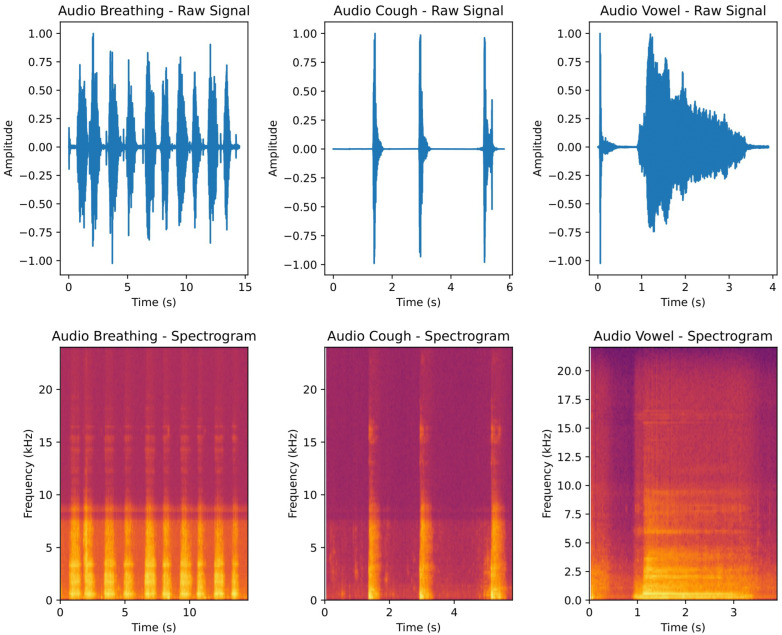
Representation of multiple audio recordings in the temporal domain (**top row**) and time-spectral domain (**bottom row**). The spectrogram representation indicates the prevalence of frequencies over specific time intervals, with lighter colours representing higher frequency preponderance.

Audios were recorded into a raw, uncompressed format (WAV, 16-bit), sampled at 16 kHz, ensuring a good quality standardisation of this procedure across different devices. After, recordings underwent a bit-depth normalisation to make them comparable under the same amplitude scale. To ensure their quality to feed further stages of the algorithm development (feature extraction, training, evaluation), a pre-trained audio classification model called YAMNet was used [21]. YAMNet is a DL architecture derived from MobileNetV1 and pre-trained on 16 kHz audios from a large audio dataset (AudioSet). It can classify audio segments into 521 audio/speech events. Specific events (breathing, cough) were used as reference points to validate our collected audio recordings by passing them through this model. Participants were only accepted if at least 50% of their audio recordings met the quality assessment criteria (i.e., fell into one of the pre-selected acceptable sound classes). Some examples of audio recordings collected from different vocalisation exercises are shown in Figure 2. In further steps, the features were only calculated for cough and breath recordings, as these were generally of better quality.

### 2.4. Algorithm Development

The algorithm designed was intended to solve a binary classification problem in which participants were classified as infected or not with the SARS-CoV-2 virus. Thus, the positive label was assigned to infected participants and the negative label to non-infected participants.

**Figure 3:**
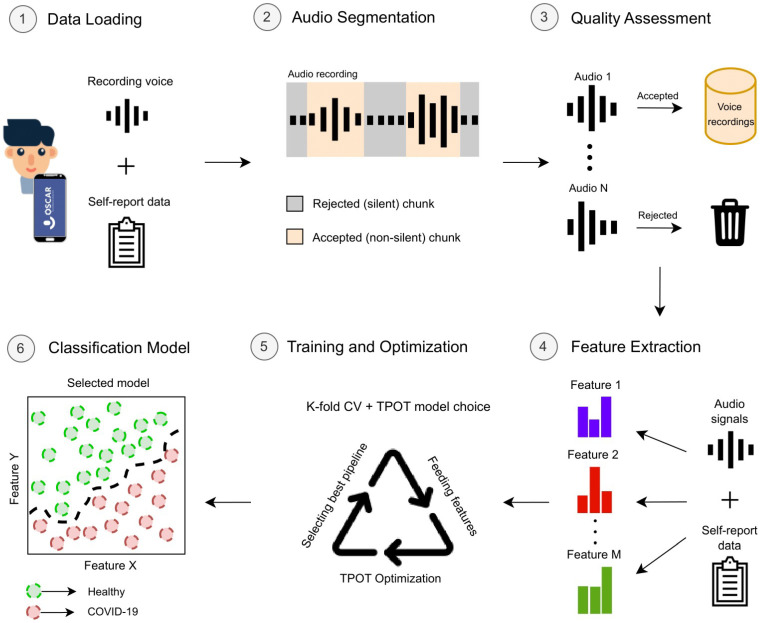
Illustration of the full data processing pipeline. It goes from the collection of audio recordings up to model training, optimisation, and evaluation.

Machine Learning (ML) aims to learn from observational data, which, in this case, comprises the participants’ voice recordings and questionnaire answers. Using signal processing expertise, all these data sources and extracted relevant characteristics from the audio signals were gathered and fed into decision-based (ML) algorithms that adjust their parameters according to the data observed during the training process. The whole data processing flow included multiple stages, namely, (a) data acquisition, (b) audio segmentation, (c) quality assessment, (d) feature extraction, (e) model training and optimisation, and (f) evaluation. Figure 3 illustrates the processing steps, after which the model is ready to evaluate data from new incoming participants. The algorithms were developed in Python language, and the pipeline (including the extracted features) was equivalent to that applied in [4], optimised using the TPOT framework [22]. Every pipeline was structured to be composed of a (i) feature scaler, (ii) feature selector, and a (iii) classification model. The TPOT’s optimisation search is conducted using genetic algorithms, involving selection, crossover, and mutation of the parameters across generations. The parameter search of scalers, selectors, and classifiers for our experiments was already defined by default in TPOT’s API [22]. Table 1 provides a detailed description of the classification parameters over which optimisation was performed during the training stage.

### 2.5. Audio Segmentation and Event Detection

Audio segmentation and segment quality assessment are essential steps in the proposed pipeline, as errors in this stage will directly propagate to the subsequent stages, particularly feature extraction and model training (e.g., speech portion found during a breathing recording will affect feature’s meaningfulness). In our data curation scenario, two quality verification filters are applied before audio segments undergo further stages, namely, (a) a non-silent segment extractor, which retrieves non-silent audio portions of a particular recording, and (b) a speech event detector that accepts each segment retrieved if it belongs to the recording it has been extracted from (e.g., breathing segments are only accepted in breathing audio files). The non-silent segment extractor is an energy-based algorithm from Librosa Python library [23], whereas the event detector is a pre-trained CNN that assigns an audio signal portion into one of (i) breathing, (ii) cough, (iii) voice, (iv) vowel-a, (v) vowel-e, or (vi) vowel-o categories. The event detector has been pre-trained in a curated subset of the Coswara dataset, achieving a macro F1-score of 84.6% [4].

### 2.6. Feature Extraction

Feature extraction is a crucial step in the processing pipeline, as it involves transforming raw audio data into a set of measurable characteristics that can be used for ML model training. After audio recordings underwent segmentation and quality assessment, features were extracted from various domains to capture the diverse aspects of the audio signals.

**Figure 4:**
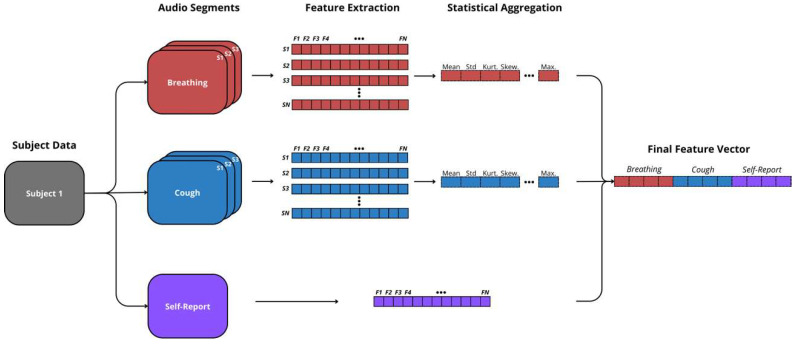
Illustration of the feature extraction pipeline. It starts with audio segments extracted by the segmenter, specifically focusing on breathing and cough sounds. The feature extraction procedure computes spectral, cepstral, temporal, amplitude, and phonetic features. These are aggregated using statistical descriptors. The final vector combines all aggregated features for breathing, cough, and self-reported data.

Each audio segment was divided into equally sized time windows, from which features were computed and averaged into a single value. The window size varied depending on the recording type, specifically, 32 ms + 50% overlap for breathing, and 16 ms + 75% overlap for cough, whose values were chosen after preliminary tests performed in [4]. Features were extracted from the (a) spectral, (b) cepstral, (c) temporal, (d) amplitude, and (e) phonetic domains of the audio segments retrieved, some of them clinically relevant from a physiological perspective (all features computed are detailed in [4]). This segment-wise approach resulted in a distinct set of features for each segment, so compatible features were aggregated using statistical descriptors (e.g., mean, std dev, kurtosis, skewness, maximum, minimum). Features from self-reported questions were simply encoded in this process. Finally, all feature domains were merged into a single vector that subsequently fed the prediction model training stage. The procedure behind the feature extraction stage is illustrated in Figure 4.

### 2.7. Training and Evaluation

**Figure 5:**
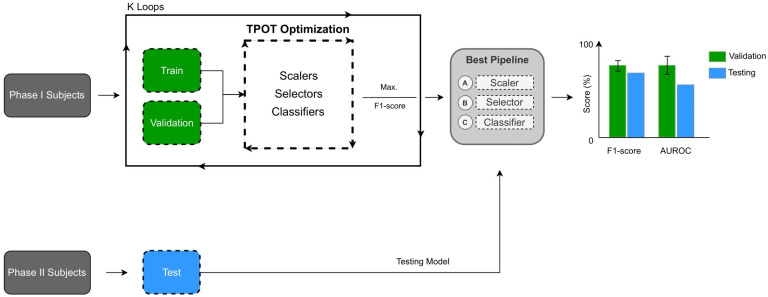
Illustration of the training, validation, and optimisation strategies. Data from Phase I subjects undergo the training loop fine-tuned by a TPOT optimisation strategy. Each pipeline (scaler, selector, and classifier) is trained and tested using K distinct combinations of patients (K = 5, 80% to train and 20% for validation). The best pipeline (maximise F1-score, macro) is selected and tested against Phase II subjects, on whom final performance is reported.

Model training and validation procedures were carried out using a K-fold cross-validation strategy [24]. This process involved an iterative training method in which the whole training set was divided into K equally sized folds of subjects. In each iteration, the model is trained with K-1 subject folds and tested on the remaining fold. The process is repeated K times to ensure the entire data set has been evaluated in distinct iterations. In our experiments, K = 5, which means that, at each iteration, 80% of the subjects were kept for training and 20% were left for validation. Finally, the results were averaged to capture the global performance scores and decide which pipeline best suits the use case. The optimisation process is illustrated in Figure 5.

The chosen pipeline was tested on Phase II participants (test set) after training and validation of the algorithm on Phase I subjects. Standard metrics were used to evaluate the model performance and translate the algorithm capabilities into the clinical context. A confusion matrix was used to represent a comprehensive overview of the model performance and confirm which classes the model predicts better or worse [25]. Multiple metric scores were then estimated from this matrix, particularly Sensitivity, Specificity, Positive and Negative Predictive Values (PPV, NPV), Accuracy, and F1-Score. The area under the receiver operating characteristic curve (AUROC) was also computed to establish a broader evaluation of Sensitivity and Specificity.

## 3. Results

A total of 322 people agreed to participate in Phase I. After quality-wise filtering, the training set was reduced by 51.6%, leaving 166 subjects effectively selected for training the classification model (34.3% SARS-CoV-2 positive). In Phase II, the test group was reduced by 52.8%, leaving 58 subjects available to evaluate the algorithm’s performance (69.0% SARS-CoV-2 positive). In Phase I, a 5-fold cross-validation strategy was employed, with the dataset being split into 80% for training and 20% for testing at each iteration. For Phase II, the pipeline selected in the previous stage was evaluated on the entire dataset (test set).

Observing the outcomes of Table 2, approximately 60.0% of the participants in both phases were female (*p* = 0.948), and the majority were under 50 years old (*p* = 0.470). The prevalent clinical symptoms reported in Phase I were flu-like, cough, fever, and sore throat. In Phase II, flu-like symptoms, cough, muscle/body aches, and headaches were the most frequent ones. A distinct symptomatic profile seemed to show up, being confirmed by the significant group differences identified in the Symptoms domain (*p*-value: 0.003). Infection rates also revealed distinct profiles, with Phase I subjects displaying a more significant proportion of non-infected subjects than Phase II (65% vs. 27%, *p*-value < 0.001).

During this first quality-wise filtering step, recordings were evaluated by the YAMNet model, and eligible subjects were moved into the next stage. Then, breathing and cough recordings were split into segments, where each was evaluated and accepted/rejected by the event detector model. After this step, the feature extraction phase was carried out, followed by model training and evaluation. During the training stage, five distinct combinations of Phase I participants were defined to iteratively fine-tune each model (subject-wise 5-fold cross-validation) [22], thus mitigating the influence of random splits in the allocation of subjects and model selection. The pipeline that achieved the highest F1-score (macro) was selected, as this score is a suitable alternative to accuracy for datasets with unbalanced classes. After this process, a Gaussian Naïve Bayes classification model, combined with a One Hot Encoder scaler and a Family-wise Error rate feature selector, provided the best results, achieving an averaged 88.2% macro F1-score (validation score). Results from the training and validation stages are provided in detail in Table 3, where ten distinct obtained pipelines are displayed in descending order of performance.

The real performance of the selected pipeline was finally assessed using a hold-out testing set (Phase II subjects). The confusion matrix depicting the results obtained from the evaluation of Phase II is shown in Table 4. Regarding performance metrics (Table 5), a sensitivity of 85.0%, specificity of 88.9%, PPV of 94.4%, NPV of 72.7%, and F1-score of 84.7% were obtained. The overall AUROC score reached 94.0%. When evaluating the same score by gender, the model achieved 87.0% for males and 97.0% for females. Considering the age factor, the model obtained 95.0%, 91.0%, 100%, 89.0%, and 100% for the age ranges of 18–29 (N = 17), 30–39 (N = 20), 40–49 (N = 8), 50–59 (N = 9), and 60–69 (N = 3), respectively. When considering the number of days between taking the SARS-CoV-2 diagnostic test and recording audio samples, the model performed less accurately when the test and recording were done on the same day (78.0%). This performance improved when audio samples were recorded one day (93%), three days (100%), and five days (89%) later.

To check the validity of the extracted audio features, we conducted a preliminary experiment using only the features extracted from the audios and discarding the self-reported data to obtain a final model. In general, the results decreased slightly, with the model misclassifying 11 out of 58 subjects when tested with Phase II subjects. Sensitivity, specificity, and F1-score reached 80.0%, 83.3%, and 79.3%, respectively.

## 4. Discussion

The development of digital voice biomarkers holds significant potential in different medical fields [26,27], as they provide non-invasive, cost-effective, and accessible means of monitoring and diagnosing health conditions. This innovative approach uses digital technology and machine learning to improve healthcare outcomes. It has already been shown to be useful in detecting cognitive impairment in preclinical and prodromal Alzheimer’s dementia [28], in Parkinson’s disease [29], as well as in identifying exacerbations of asthma [30,31,32] and chronic obstructive pulmonary diseases [33].

Different research groups have tried to develop more convenient and accessible methods to diagnose COVID-19 [7]. One of the methodologies used is the analysis of voice recordings using machine learning to design predictive algorithms [12,34,35]. It is anticipated that these algorithms can be used as a screening tool that any person can use before accessing a clinical facility asking for a common antigen or RT-PCR test.

Even though the World Health Organisation no longer considers COVID-19 a global health emergency, it is still crucial for States Parties to consider how they can improve their preparedness for future outbreaks. In this sense, continuous research is needed to find easily implementable detection methods [36].

In Phase I, we developed an algorithm for screening COVID-19-compatible symptoms using a real-world set of community-based participants from the population. Sensitivity and specificity were two clinically relevant metrics to evaluate the model’s performance in the test group (Phase II). Consequently, we expected the model to be sensitive to COVID-19 cases and specific enough not to classify all cases as COVID-19. Specificity should, therefore, ensure that a minimum number of negative cases (without COVID-19) are classified as COVID-19 (false positive).

It is worth noting that the two recruitment phases were conducted at different times, and different SARS-CoV-2 variants prevailed. In Phase I, the Delta variant was most prevalent in Portugal, while in Phase II, a higher proportion of Omicron-linked cases were reported. Table 2 shows that although gender and age appeared to be similarly distributed in both phases, there were significant differences in symptomatic profiles. Although the model was developed based on Phase I data, it was still able to distinguish well enough between infected and uninfected participants in Phase II. This could give rise to the hypothesis that the vocal characteristics of COVID-19 patients may be stable across different virus variants.

Our results show that the algorithm provides relevant insights by exhibiting appropriate behaviour and misclassifying only 8 out of 58 subjects available for testing. Furthermore, it shows adequate sensitivity in identifying positive and negative cases (85.0% sensitivity, 88.9% specificity) and a high positive predictive value (up to 94.4%). These results suggest that the model predicts positive SARS-CoV-2 cases more reliably than negative cases (considering the lower negative predictive value of 72.7). Gender differences led to a change in the model’s AUROC values of about 10%, with the female group performing better than the male group. This fact should be considered in future studies with regard to the need for separate models to account for such differences.

Regarding age differences, the AUROC scores did not show significant variations, except for a slight decrease in performance observed in the 50–60 age range. COVID-19 fatality rates are much higher in older people than in the youth population [37]. If age-related factors influence the model’s decision-making process, priority and increased importance should be given to older age groups during algorithm development, considering their lower likelihood of survival.

When looking at the time between performing the test and recording the audio samples, the model performed better the more days passed between the two events. This may indicate that the features capture symptom patterns that do not appear or intensify until several days after diagnosis [38]. This can have practical implications for the timing of the diagnosis, as a substantial part of the SARS-CoV-2 virus transmission occurs during the presymptomatic phase [39]. However, a more comprehensive validation using a larger data set would help to confirm and strengthen all hypotheses and results, and to better understand the biological mechanisms behind these patterns.

The model’s performance also decreased when the self-reported data were removed from the feature set (84.7% to 79.3% macro-F1 score), although it still showed appropriate behavior. This suggests that subjects’ self-assessment may complement the audio-based patterns, which may not be able to identify some difficult cases (e.g., already with symptoms but without manifestations on the vocal tract).

In terms of performance, asymptomatic participants (with symptoms but not infected) may also explain the model’s worse performance, as we found more misclassified COVID-19-infected than non-infected individuals. Even so, the model achieved a macro F1-score of 84.7%, accuracy of 86.2%, and AUROC of 94.2%, which is remarkable. Even though most studies described previously [12,13,14] achieved notable performance scores (AUROC ranging from 84.6% to 98.6%), those also relied on their training and evaluation strategies in crowdsourced, non-curated audio data obtained from uncontrolled settings and associated with non-validated SARS-CoV-2 labels, which might have masked the validity of potential outcomes and the achievement of identical performance scores. Alternative studies conducted by Robotti et al. and Costantini et al. [17,18] designed and conducted their own data collection protocol, revealing more consistent outcomes with optimal AUROC scores of 94.0% obtained against a hold-out testing set of 30 and 36 subjects, respectively. Both the performance (94.0% vs. 94.2%) and the test set size (30/36 vs. 58) were similar and comparable, suggesting consistency across the three studies, all of which designed a precise and controlled data collection protocol with validated labels as ground truth. Some differentiating factors of our study, such as the meticulous data collection, the data curation procedure (quality analysis, segmentation, and event detection), and the use of validated SARS-CoV-2 tests to target participants as positive or negative, might have contributed to obtaining such results and definitely supported the validity of these findings.

From an ethical perspective, the use of voice data and algorithms in healthcare requires careful consideration of patient autonomy and consent. Voice data is inherently personal and can reveal sensitive information. For this reason, robust encryption and data protection protocols are needed to prevent unauthorised access or misuse. Moreover, regulations such as the GDPR in Europe impose strict requirements for the collection, storage, and sharing of personal data. The AI Act regulation will also require accountability and transparency on the limitations, misuses, and validation of predictive models used in clinical practice. Such regulations will help ensure the protection of patient data and safety of AI systems upon their usage in real-world settings.

In future work, validation in a larger population group should be performed to prove the efficiency of the approach at a national scale. Our testing set does not fully represent the target population, so performance is expected to change as long as the sample size increases. Additionally, in a follow-up study, our team plans to assess the contribution of each data source to the model’s performance using ablation techniques. This will help identify the most important features that could serve as potential digital biomarkers for COVID-19 screening or broader respiratory health assessments. Moreover, such endpoints might also reveal links between underlying biological mechanisms in the vocal tract, symptoms, and characteristics of vocal sounds, being of great interest to inform the medical community, in particular, pulmonology specialists.

## 5. Conclusions

Voice screening algorithms offer an attractive approach to assess respiratory diseases, including COVID-19. This study demonstrates a rapid and convenient method that has the potential to improve the screening strategy to identify positive cases and reduce disease transmission. With sensitivity and specificity rates of 85.0% and 88.9%, respectively, the algorithm correctly identifies most cases, so its performance in a real-world scenario is promising.

One of the key findings of this study is the potential for expansion beyond COVID-19, where identical approaches could be explored to monitor and characterise other respiratory diseases such as asthma or chronic obstructive pulmonary disease (COPD), as well as respiratory infections like seasonal influenza and tuberculosis. The easy accessibility and simplicity of such a data collection method offers significant advantages for extending such approaches to any clinical unit, as audio recordings are non-invasive, inexpensive, and easy to collect.

Our findings highlight the promising potential of voice-based screening strategies, but also leave room for future research in the field of audio signal processing in combination with ML as a complementary method for screening respiratory diseases, especially in controlled clinical settings. Further research should focus on refining such algorithms and processing pipelines, investigating explainability techniques to ensure clinical interpretability of the results, and validating outcomes in large population cohorts to better account for limitations and the impact of specific sources of bias (e.g., gender, age, ethnicity) and maximise clinical utility. Advances in this area will improve diagnostic procedures and better support early intervention in patients.

## Figures and Tables

**Table 1:** Description of the parameter search space for classification models considered by TPOT. Parameters were based on TPOT’s default ranges and used to select the best-performing classification model for the proposed binary classification task.

Model	Parameters	Search Range
NB	alpha	{1 × 10^−3^, 1 × 10^−2^, 1 × 10^−1^, 1, 10, 100}
	fit_prior	{True, False}
Tree-based(RF, ET, GB, XGB, DT)	criterion	{“gini”, “entropy”}
	max_depth	[1, 11]
	max_features	[0.05, 1.0]
	min_samples_split	[2, 21]
	min_samples_leaf	[1, 21]
	min_child_weight	[1, 21]
	learning_rate	{1 × 10^−3^, 1 × 10^−2^, 1 × 10^−1^, 0.5, 1}
	subsample	[0.05, 1]
	bootstrap	[True, False]
KNN	n_neighbors	[1, 101]
	weights	{“uniform”, “distance”}
	p	{1, 2}
SVM	penalty	{“l1”, “l2”}
	loss	{“hinge”, “squared_hinge”}
	dual	{True, False}
	tol	[1 × 10^−5^, 1 × 10^−1^]
	c	[1 × 10^−4^, 25]
LR	penalty	{“l1”, “l2”}
	c	[1 × 10^−4^, 25]
	dual	{True, False}
SGD	loss	{“log”, “hinge”, “modified_huber”, “squared_hinge”, “perceptron”}
	penalty	{“elasticnet”}
	alpha	{0, 1 × 10^−1^, 1 × 10^−2^}
	learning_rate	{“invscaling”, “constant”}
MLP	alpha	{1 × 10^−4^, 1 × 10^−3^, 1 × 10^−2^, 1 × 10^−1^}
	learning_rate_init	{1 × 10^−3^, 1 × 10^−2^, 1 × 10^−1^, 0.5, 1}

NB—Naïve Bayes; RF—Random Forest; ET—Extra Trees, GB—Gradient Boosting, XBG—eXtreme Gradient Boosting; DT—Decision Tree; KNN—K-Nearest Neighbors; SVM—Support Vector Machine; LR—Logistic Regression; SGD—Stochastic Gradient Descent; MLP—Multi-Layer Perceptron.

**Table 2:** Population characterisation by gender, age, and symptoms, regarding both Phase I and Phase II groups.

Demographic and Clinical Data		Distribution by Groups		*p*-Value ^a^
		Phase I (N = 166)	Phase II (N = 58)	
Gender	Female	99	35	0.948
	Male	66	23	
	N.A.	1	0	
Age (years)	18–29	70	17	0.470
	30–39	30	20	
	40–49	35	8	
	50–59	21	9	
	60–69	8	3	
	70–79	2	1	
Symptoms ^b^	Flu-like	49	35	0.003 *
	Cough	45	33	
	Fever	33	12	
	Sore throat	33	19	
	Body aches/myalgias	30	32	
	Headaches	27	23	
	Loss of smell/taste	18	1	
	Shortness of breath	8	5	
	Diarrhoea	8	1	
SARS-CoV-2	Infected	57	40	<0.001 *
	Not Infected	109	18	

N.A.: Not answered; *—significance value at 5%; ^a^—*p*-values were obtained using a non-parametric one-way ANOVA/Chi-squared test; ^b^—multiple-choice items (does not total 100%).

**Table 3:** Ranking of top 10 pipelines returned by TPOT using data from Phase I participants. F1-score (macro average) was defined to establish the pipeline selection criteria score. Scores are displayed in descending order and results from an average over five distinct subject train/test folds. The best-performing and selected pipeline is bolded and underscored.

Dataset	Rank	TPOT Pipeline			F1-Score (%) *
		Scaler	Selector	Classifier	
OSCAR	**1**	**OneHot**	**FwE**	**Gauss. NB**	**88.2**
	2	OneHot	FwE	Multi. NB	86.3
	3	Binarizer	Var. Thresh	SVM Linear	84.5
	4	Polynomial	Percentile	Extra Trees	83.7
	5	Robust	Var. Thresh	Random Forest	80.1
	6	RBF Sampler	FwE	SVM Linear	74.3
	7	RBF Sampler	FwE	MLP	70.5
	8	Max. Abs.	FwE	Extra Trees	66.4
	9	Robust	FwE	KNN	60.5
	10	Robust	RFE	MLP	52.9

*—F1-score (macro) value from a 5-fold average of distinct participant splits; Max. Abs.—Maximum Absolute scaler; One Hot—One Hot Encoder; Polynomial—Polynomial feature generation; Var. Thresh—Variance Threshold; Percentile—Percentile selector; FwE—Family-wise Error rate selector; RFE—Recursive Feature Elimination; MLP—Multi-Layer Perceptron; Gauss. NB—Gaussian Naïve Bayes; Multi. NB—Multinomial Naïve Bayes; KNN—K Nearest Neighbors.

**Table 4:** Classification model raw performance on Phase II testing set. The confusion matrix reports the raw behaviour of the Naïve Bayes algorithm trained on Phase I subjects and tested on Phase II participants.

		Expected	
		COVID-19	Without COVID-19
Predicted	COVID-19	34	2
	Without COVID-19	6	16

**Table 5:** Classification model performance scores on Phase II testing set. In this refined version, seven evaluation metrics are computed and reported (%) to capture a global perspective of the Naïve Bayes model performance.

Dataset			Performance Metrics (%)				
	Sensitivity	Specificity	PPV	NPV	F1-Score *	AUROC	Accuracy
OSCAR Dataset	85.0	88.9	94.4	72.7	84.7	94.0	86.2

*—macro score; PPV—positive predictive value; NPV—negative predictive value; AUROC—area under the ROC curve.

## Data Availability

Data not available due to ethical restrictions.
